# Decreased Information Replacement of Working Memory After Sleep Deprivation: Evidence From an Event-Related Potential Study

**DOI:** 10.3389/fnins.2019.00408

**Published:** 2019-04-26

**Authors:** Liwei Zhang, Yongcong Shao, Zhongqi Liu, Chenming Li, Yuhong Chen, Qianxiang Zhou

**Affiliations:** ^1^School of Biological Science and Medical Engineering, Beihang University, Beijing, China; ^2^Beijing Advanced Innovation Centre for Biomedical Engineering, Beihang University, Beijing, China; ^3^Cognitive and Mental Health Research Center, Beijing Institute of Basic Medical Science, Beijing, China; ^4^The Quartermaster Research Institute of Engineering and Technology, Beijing, China

**Keywords:** working memory, sleep deprivation, information replacement, event related potential, electroencephalography

## Abstract

Working memory (WM) components are altered after total sleep deprivation (TSD), both with respect to information replacement and result judgment. However, the electrophysiological mechanisms of WM alterations following sleep restriction remain largely unknown. To identify such mechanisms, event-related potentials were recorded during the n-back WM task, before and after 36 h sleep deprivation. Thirty-one young volunteers participated in this study and performed a two-back WM task with simultaneous electroencephalography (EEG) recording before and after TSD and after 8 h time in bed for recovery (TIBR). Repeated measures analysis of variance revealed that, compared to resting wakefulness, sleep deprivation induced a decrease in the P200 amplitude and induced longer reaction times. ERP-component scalp topographies results indicated that such decrease primarily occurred in the frontal cortex. The N200 and P300 amplitudes also decreased after TSD. Our results suggest that decreased information replacement of WM occurs after 36 h of TSD and that 8 h TIBR after a long period of TSD leads to partial restoration of WM functions. The present findings represent the EEG profile of WM during mental fatigue.

## Introduction

Working memory (WM) is a cognitive function with limited capacity (Miyake and Shah, 1999), and is used to store and process information. It provides a temporary storage space and the information necessary for processing tasks, such as speech understanding, reasoning, and learning. WM is a bridge between the instantaneous and long-term memory systems and is important for information processing in humans (Baddeley, 2000). According to Baddeley’s model, WM consists of the central executive, visuospatial sketchpad, phonological loop, and episodic buffer (Baddeley and Hitch, 1974; Baddeley, 2000). The information entering the phonological loop and visuospatial components is processed by the centralized control of the central executive system.

The neural mechanism of WM based on Baddeley’s model has been extensively discussed. According to differences in information processing, WM is divided into different types, such as phonological, spatial, and object WM. Most researchers believed that phonological WM was confined to the posterior parietal cortex and the frontal region of the left hemisphere (Brodmann’s areas, premotor area, and auxiliary motor area). However, the frontal-parietal region activity did not exclude the contribution of word information storage to language information processing (Awh et al., 1996; Wegrzyn et al., 2017). Researchers investigating the brain structures involved in spatial WM assumed that the posterior dorsal parietal lobe (Brodmann area 40) was involved in the storage of spatial WM and emphasized the role of the frontal lobe in spatial-information processing (Pierard et al., 2011), as the brain region activated by object WM tasks overlaps with this structure. Moreover, the activation intensity of the dorsolateral prefrontal cortex increased with the WM load (Michael et al., 2006; Lythe et al., 2012). When studying WM using the n-back test paradigm, the dorsolateral prefrontal cortex (Brodmann area 9/46) and anterior cingulate gyrus (Brodmann area 24/32) were often observed to be active. The n-back task is based on the reciprocal n-test paradigm and is one of the most commonly used cognitive experiments on WM (Owen et al., 2005; Jaeggi et al., 2010). During the n-back task, when *n* = 0, subjects were asked to compare the current stimulus with the first stimulus; when *n* = 1, subjects were asked to compare the current stimulus with the immediately preceding stimulus.

The neural mechanism of WM involves the cortical circuits connecting the prefrontal cortex to the posterior parietal cortex (Smith and Jonides, 1995; Owen et al., 1996), and this mechanism can be observed using electroencephalography (EEG) signals in humans. A close relationship exists between the contingent negative variation level in the prefrontal and temporal information processing. Specifically, the negative slow wave in the left frontal region and the bilateral positive slow waves in the parietal region reflect the function of phonological WM, while a spatial WM task triggers negative slow waves in the posterior parietal region (Mecklinger and Pfeifer, 1996). At present, the most studied component of the WM is the P300 wave. Donchin and Fabiani (1991) demonstrated that the P300 wave is induced by the updating of WM content and decreases with increased WM load. Beydagi et al. (2000) systematically investigated the relationship between the amplitude and latency of ERPs and WM recall time. The results revealed that increased recall time significantly increases the latency of the P300 wave, while the early components of ERP related to WM, such as the N100, P200, and N200 waves did not correlate with the latency or amplitude of memory (Beydagi et al., 2000). The neural source of the P200 is not yet known, but some evidence indicates that this wave may reflect general neural processes that occur when a visual (or other sensory) input is compared with an internal representation or expectation in memory or language (Evans and Federmeier, 2007).

Sleep deprivation is a common social phenomenon that impairs distracter suppression (Eve et al., 2018) and human WM functions (Blasiman and Was, 2018). The effects of sleep deprivation on short-term WM are reflected in basic attention and information processing. Sleep deprivation leads to impairment of neurotransmitter receptors in the brain regions involved in memory (Pierard et al., 2011), such as the hippocampus, and affects the consolidation and reconsolidation of memory processes (Stickgold, 2005). Drummond et al. (2000) used functional magnetic resonance imaging (fMRI) to evaluate the effect of 35 h of sleep deprivation on the brain activation of young individuals using verbal tasks, and found that the excitability of the temporal and bilateral parietal lobes increased. However, other reports have indicated a decrease in the activation of the parietal cortex after sleep deprivation (Almklov et al., 2015). Moreover, the results revealed that the decrease in activation was significantly correlated with a decrease in short-term memory after sleep deprivation (Adrienne, 2013; Xie et al., 2015; Nicolas et al., 2016; Julien et al., 2017). Previous study using an n-back task to test WM in participants who had been sleep-deprived for 24 h found that sleep deprivation leads to a reduction in metabolic activity in the brain’s regional network (prefrontal cortex, anterior cingulate gyrus, thalamus, and cerebellum) mainly involved in information processing and executive control (Choo et al., 2005).

Although attempts are made to maintain wakefulness and performance, sleep deprivation will affect the information processing ability of WM and reduce processing speed (Casement et al., 2006). A high temporal resolution EEG study found that after sleep deprivation, the functional connectivity of the prefrontal cortex was strongly affected, and the aggregation coefficient (local integral) decreased in the alpha (α) frequency band; however, the path length (global integral) increased in the theta (θ) band (Verweij et al., 2014). Moreover, Cajochen et al. (2000) found that the power densities of the δ and θ bands activated by the frontal EEG were significantly higher than those of the parietal and occipital regions after 40 h of sleep deprivation in young healthy participants. Studies on sleep deprivation identified P300 wave as a potential biomarker for sleep deprivation (Morris et al., 1992; Kusztor et al., 2019). The latency of the P300 wave in the central and frontal regions was significantly delayed and the amplitude of the P300 wave in the central region exhibited the greatest decrease (Cote et al., 2009; Matthyssen, 2013). Some researchers believed the decrease in the P300 wave amplitude to be caused by the reduction in cognitive resources used to evaluate stimulation (Matthyssen, 2013), while others believed that the P300 and other slow waves reflected the effect of performance inhibition (Brauer, 2016). Moreover, after sleep deprivation, the amplitude of the N100 wave, which is reflective of early sensory processing, was decreased, and its latency was increased. These changes reflect the slowing of the sensory coding process or delays in the attention to process (Brauer, 2016).

Although a significant number of studies have investigated the ERPs, research has not been systematically conducted, and the results have not been consistent (Evans and Federmeier, 2007; Eve et al., 2018; Waninger et al., 2018). A study has reported that the P200 wave and the late positive complex are sensitive to changes in task attention and WM requirements (Smith et al., 2002). However, there are few studies on EEG signals for the P200 wave in sleep deprivation (Lee et al., 2004; Mograss et al., 2009). The electrophysiological changes related to WM processing before and after sleep deprivation, including the changes in early and late components of ERP after restorative sleep, have rarely been systematically studied. In a combined behavioral and ERP experimental design, we used the two-back WM task paradigm to explore the changes in brain ERP after 36 h of sleep deprivation and 8 h TIBR. There were two hypotheses: (1) After sleep deprivation, WM performance decreases, and the amplitude of the EEG N200 and P300 waves also decreases, as they reflect the central executive control in WM; and (2) 8 h TIBR has a restorative effect on WM, reflected in the recovery of these ERP indices.

## Materials and Methods

### Participants

Thirty-one postgraduate men from Beihang University participated in this study. The selected participants were right-handed and maintained good sleeping habits. All participants were 23–27 years of age (mean = 24.6, standard deviation = 2.01), had no history of neurological or psychiatric disorders or somatic disease, used English as their second language, and had no special training in memory skills. All participants had a normal or corrected-to-normal vision, >110 intelligence scores on the Raven Test, and provided written informed consent. The study was conducted according to the principles of the 1964 Declaration of Helsinki. The ethics committee of the Beihang University approved this study. Subjects needed to get in bed before 10 pm and get up after 6 am each day with 7–9 h’ sleep. All participants indicated that they had maintained regular sleep patterns for >1 week and did not smoke, drink alcohol, drink coffee, or consume any medication for 48 h prior to the experiment. Before the test, they were thoroughly acquainted with the experimental methods and procedures.

### Stimuli Presentation

**FIGURE 1:**
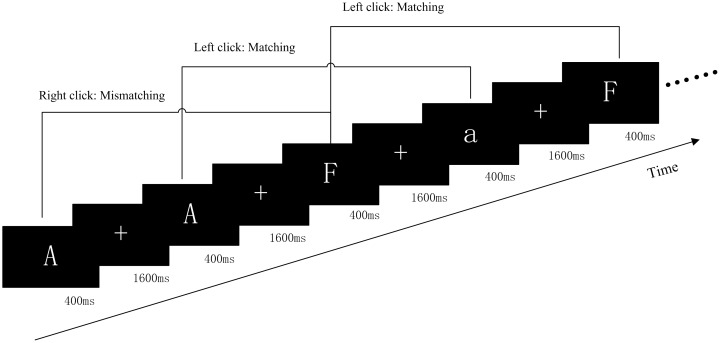
Schematic diagram of the PWMTB tasks. Participants were instructed to remember the various letters of an array consisting of 15 letters. There were two blocks of PWMTB tasks, and each block consisted of 60 trials. The target was presented for 400 ms. Participants were instructed to press the mouse button as rapidly as possible.

Participants were instructed to complete a classical pronunciation WM two-back task (PWMTB), illustrated in Figure 1. The task was coded using the E-prime software. The stimuli were 15 English letters (a, d, e, f, g, r, t, A, D, E, F, G, R, T, and H) excluding letters with similar glyphs, such as L, l, M, and m, and the stimuli were not case sensitive. These letters, presented in white color on a black background, subtended an approximate visual angle of 4° × 4° (width: 2.0 cm, height: 2.0 cm). In each trial, the target was presented for 400 ms, followed by a black screen for 1600 ms, and the participants matched the current letter with the letter presented two trials prior, to determine whether the pronunciation was consistent. If the pronunciation was the same (“Matching”), then the left mouse button was pressed; otherwise (“Mismatching”) the right mouse button was pressed. “Matching” and “Mismatching” conditions were presented in a pseudo-random order with a 1:1 ratio. Each run consisted of two blocks of 60 trials and each block lasted 120 s. The target was presented using a 21-inch monitor (refresh rate: 60 Hz) at a 70 cm viewing distance. To prevent the effects due to memorization, three different orders for the letters were used.

### Experimental Procedures

**FIGURE 2:**
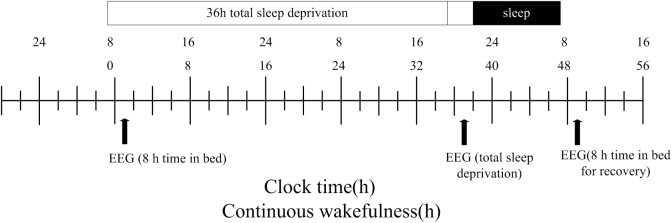
Study design. After 8 h time in bed (TIB), subjects underwent total sleep deprivation for 36 h followed by recovery sleep for 8 h. The black arrows indicate the time points of the EEG recordings.

The participants completed two visits to the laboratory. The first visit involved a simple session during which they were informed of the experimental methods. They were then instructed to practice the PWMTB task until an accuracy rate of 80% was achieved. Three EEG recordings were conducted in the second session (Figure 2). All participants underwent sleep monitoring from the first night onward, and the PWMTB task was performed at 8 am the following morning with simultaneous EEG recording. The second EEG recording was conducted after a 36 h period during which the participants were not allowed to sleep. The participants then slept for 8 h, after which the third EEG recording was completed. The participants were only allowed to perform non-strenuous activities during the 36 h period, such as conversing, reading, gaming, and working on a computer. Moreover, participants were not permitted to smoke, drink, or consume any stimulants including coffee, chocolate, soft drinks, or alcohol.

### EEG Data Collection and Recordings

Electroencephalography scalp recordings were acquired using the Scan 4.5 software (Neuroscan Products) using a SynAmps2 amplifier (Neuroscan Products). EEG data were continuously recorded using Quik-Cap with 32 scalp electrodes (Neuroscan Products) positioned over the entire scalp according to the 10–20 system. The sampling frequency was 1000 Hz, and the electrode impedances were maintained below 5 kΩ. The reference electrodes were placed on the bilateral mastoids.

### Data Analysis of Behavioral Experiments

All data on behavioral experiments in the “Matching” and “Mismatching” conditions were restricted to trials with response times (RT) >300 ms and <2000 ms. Behavioral data recorded in the 8 h TIB, TSD, and TIBR states were statistically tested via repeated measures analysis of variance (ANOVA), with Greenhouse-Geisser correction for non-sphericity and Bonferroni *post hoc* analysis. Statistical tests were carried out using IBM SPSS Statistics software (V 22.0). Data are represented as mean and standard deviation (SD).

### EEG Data Analysis

The raw EEG data were analyzed offline using Scan 4.5. The EEG eye movement artifacts were corrected using the ocular artifact reduction method as implemented in Scan 4.5. Epochs with a length of 1000 ms ranging from -200 ms to 800 ms with respect to the onset of the stimuli were then extracted from the continuous EEG data. Trials with incorrect responses or RT outside the acceptable time range (300–2000 ms) were excluded. The stimuli-locked ERP was baseline-corrected in the range of -200 ms to 0 ms before stimuli onset. Automatic artifact reduction was implemented to inspect the epoch data. The block interval ranged from -100 ms pre-artifact to 100 ms post-artifact, and the amplitude was between -100 and 100 μV. A band-pass filter from 0.5 to 40 Hz was then used to filter the epoch data. The frequency slope of the filter was 24 dB/oct. Stimuli-locked data averages were computed separately for each participant and each state.

The ERP components P200 (120–200 ms), N200 (200–350 ms), and P300 (300–450 ms) of the stimulus trials were identified and quantified. The grand-average peak amplitudes and latencies of the three components were calculated separately at F3, Fz, F4, C3, Cz, C4, P3, Pz, and P4. These areas have been shown to display altered activation following sleep deprivation (Casement et al., 2006; Verweij et al., 2014).

All ERP results were statistically analyzed using repeated measures ANOVA, which revealed the main effects and the interactions between sleep states (TIB, TSD, and TIBR), regions (frontal, central, and parietal), and lines[left (F3, C3, and P3), middle (Fz, Cz, and Pz), and right (F4, C4, and P4)]. ANOVA included Greenhouse-Geisser corrections for non-sphericity and Bonferroni *post hoc* tests. For the ERP-component scalp topographies display, a paired *t*-test was used to determine whether the difference between the peak amplitudes of the TIB and TSD states was significant. The same procedure was applied to the peak amplitudes of the TIB and TIBR states. Data are represented as mean and standard deviation (SD).

## Results

### Results of Behavioral Experiments

The results of the behavioral experiments are shown in Table 1. The RT in the Matching condition trials was longer in the TSD state, with a tendency to increase, but without significant differences. The trend for accuracy was opposite to that of RT, exhibiting a tendency to decrease in the TSD state. The trend for missed time was the same as RT, with no significant differences. After 8 h-TIBR, the level of RT and missed time were restored. The results of the Mismatching condition trials exhibited the same trend as those of the Matching condition trials. ANOVA revealed that the accuracy of the Mismatching condition trials was significantly different between the 36 h-TSD and 8 h-TIBR states [*F*(2,28) = 5.761, *p* = 0.017] and that the missed time of the Mismatching condition trials was significantly different between the 36h-TSD and 8 h-TIB states [*F*(2,28) = 4.347, *p* = 0.024].

**Table 1:** Performance data (mean ± SD) on the two-back tasks during 8 h time in bed (TIB), after 36 h of total sleep deprivation (TSD), and after 8 h time in bed for recovery (TIBR).

		8 h-TIB	36 h-TSD	8 h-TIBR
**Matching**	RT (ms)	541.0 (101.5)	573.4 (158.1)	510.9 (81.0)
	Accuracy (%)	92.5 (6.6)	86.0 (11.4)	91.0 (5.8)
	Missed (time)	0.846 (1.287)	2.654 (4.604)	0.769 (1.818)
**Mismatching**	RT (ms)	608.2 (120.7)	619.5 (166.1)	543.1 (69.6)
	Accuracy (%)	94.2 (5.0)	87.1 (11.6)	93.9 (4.1)^#^
	Missed (time)	0.462 (1.140)	3.231 (5.046)^∗^	1.346 (2.591)


*^∗^36 h-TSD vs. 8 h-TIB, *p* < 0.05; ^#^8h-TIBR vs. TSD, *p* < 0.05; SD: Standard Deviation*.

### Results of ERP Recordings

Cognitive decline was observed after TSD. However, after TIBR, the level of cognitive function was restored to a certain extent. The ERP results are presented using three main subsections: P200, N200, and P300. Matching and Mismatching conditions are presented within each subsection.

#### P200

##### Matching stimuli

**FIGURE 3:**
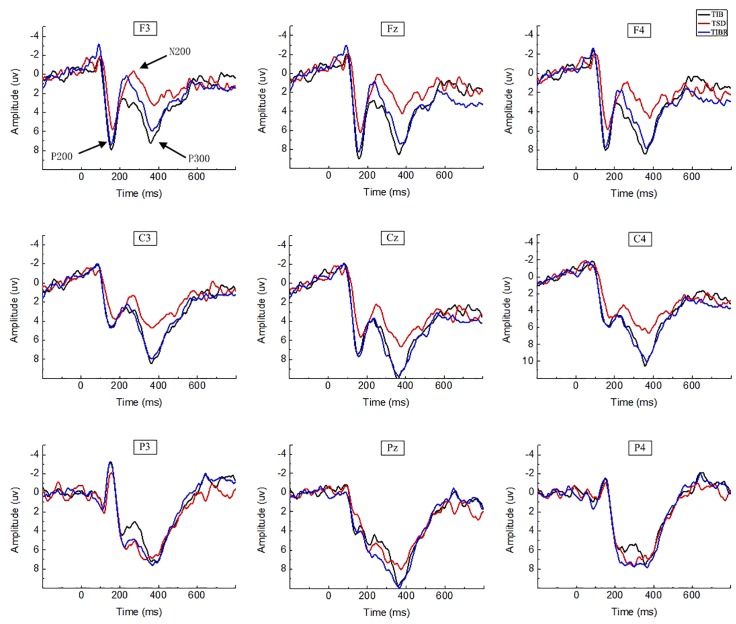
ERP amplitude between sleep conditions for Matching stimuli of working memory. Grand-average ERP (P200, N200, and P300) in the Matching stimuli condition across multiple electrode sites after 8 h time in bed (TIB), after 36 h of total sleep deprivation (TSD), and after 8 h time in bed for recovery (TIBR). The channels are ordered left-right and top-bottom as follows: F3, Fz, F4, C3, Cz, C4, P3, Pz, and P4.

**FIGURE 4:**
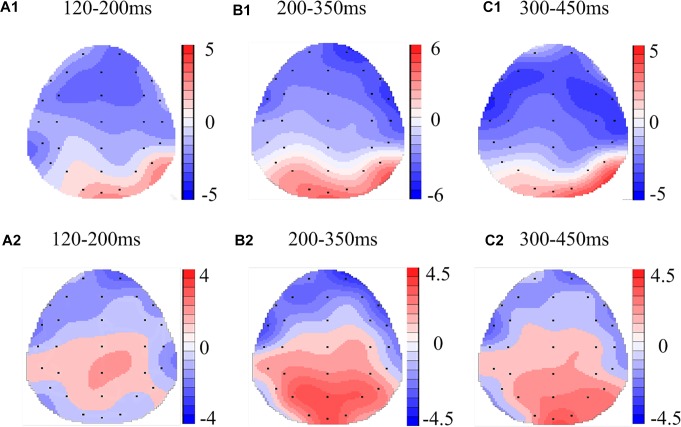
Topographic map of the differences between sleep conditions for Matching stimuli of working memory **(A1–C2)**. Head plots of the paired *t*-test approach (two-tailed *t*-test, *p* < 0.05) map the scalp distribution of statistical differences of 36 h of total sleep deprivation (TSD) vs. 8 h time in bed (TIB) **(A1–C1)** and 8 h time in bed for recovery (TIBR) vs. TIB **(A2–C2)**. Color bars indicate *t*-values. **(A1)** P200, 120–200 ms, in TSD vs. TIB. **(B1)** N200, 200–350 ms, in TSD vs. TIB. **(C1)** P300, 300–450 ms, in TSD vs. TIB. **(A2)** P200, 120–200 ms, in TIBR vs. TIB. **(B2)** N200, 200–350 ms, in TIBR vs. TIB. **(C2)** P300, 300–450 ms, in TIBR vs. TIB.

In the matching condition, the P200 wave occurred 120–200 ms after stimulus onset in our recordings, and represented the frontal region (Figure 3, F3, Fz, F4). The topographic maps of statistical differences in ERP activity between the TSD and TIB states revealed a significant decrease (most significant: Fz channel, two-tailed paired *t*-test, *t* = 3.957, *p* < 0.05) in cortical activity, primarily in the frontal regions (Figure 4A1) and between the TIBR and TIB states had no significant differences (Figure 4A2).

The main effects of sleep state [*F*(2,28) = 10.375, *p* < 0.003], region [*F*(2,28) = 32.356, *p* < 0.001], and line [*F*(2,28) = 10.894, *p* < 0.001] were significant for the P200 peak latency. These effects were qualified by a significant sleep state × region interaction [*F*(4,26) = 3.802, *p* < 0.045], indicating that the P200 responses in the frontal region increased in peak latency from the TIB to the TSD state in the Matching condition (Table 2). Bonferroni *post hoc* analysis revealed that the peak latency was significantly longer in the TSD state than in the TIB state (*p* < 0.05).

**Table 2:** Grand-average peak latency of the P200, N200, and P300 components in the Matching stimuli condition across multiple electrode sites (F3, Fz, F4, C3, Cz, C4, P3, Pz, and P4) during 8 h time in bed (TIB), after 36 h of total sleep deprivation (TSD), and after 8 h time in bed for recovery (TIBR).

		8 h-TIB			36 h-TSD			8 h-TIBR		
		P200	N200	P300	P200	N200	P300	P200	N200	P300
F3	Mean	162.7	251.8	361.8	171	263.2	381.3	161.8	240.1	383.4
	(SD)	(20.4)	(39.2)	(38.3)	(23.6)	(41.6)	50.6)	(15.5)	(34.4)	(51.0)
Fz	Mean	162.2	246.3	352.1	171.8	256.9	380.1	162.9	237.1	383.7
	(SD)	(19.0)	(40.3)	(26.0)	(23.5)	(41.3)	(35.4)	(15.9)	(34.6)	(48.8)
F4	Mean	173.4	235.3	344.36	178.5	260.5	375.1	165.3	230.5	374.3
	(SD)	(29.3)	(33.3)	(30.0)	(25.1)	(37.3)	(40.0)	(21.0)	(23.8)	(33.5)
C3	Mean	174.7	255.5	350.1	181.6	266.8	383.1	172.5	239.3	360.4
	(SD)	(34.3)	(31.1)	(45.2)	(32.1)	(30.5)	55.5)	(26.1)	(26.6)	(23.3)
Cz	Mean	165.8	252.5	348.5	183.2	252.3	387.1	169.9	233.2	351.7
	(SD)	(23.4)	(29.7)	(22.5)	(33.4)	(32.3)	55.7)	(36.4)	(22.9)	(27.6)
C4	Mean	167.3	241.3	347.5	182.3	238.5	367.6	168.9	228.9	351
	(SD)	(26.8)	(32.8)	(22.0)	(31.1)	(37.6)	40.8)	(24.8)	(27.5)	(28.1)
P3	Mean	207.1	272.8	366.5	202.5	253.5	362.6	224.3	280.2	362.6
	(SD)	(20.0)	(31.2)	(31.5)	(33.0)	(40.4)	47.2)	(21.5)	(30.1)	(30.6)
Pz	Mean	181.3	262.7	352.9	202	266.2	372.3	203	271.2	361.1
	(SD)	(29.8)	(35.5)	(22.8)	(30.2)	(42.7)	49.2)	(38.2)	(42.5)	(22.6)
P4	Mean	192	267.8	350.9	239.8	281.2	342.9	241.7	282	358.9
	(SD)	(40.5)	(54.0)	(25.0)	(39.5)	(51.5)	30.3)	(28.2)	(43.4)	(30.3)


The line main effect was significant [*F*(2,28) = 12.199, *p* < 0.001] for the P200 peak amplitude, indicating the occurrence of larger P200 amplitudes in the central than in the frontal (*p* < 0.001) and parietal regions (*p* < 0.05, see Table 3).

**Table 3:** Grand-average peak amplitude of the P200, N200, and P300 components in the Matching stimuli condition across multiple electrode sites (F3, Fz, F4, C3, Cz, C4, P3, Pz, and P4) during 8 h time in bed (TIB), after 36 h of total sleep deprivation (TSD), and after 8 h time in bed for recovery (TIBR).

		8 h-TIB			36 h-TSD			8 h-TIBR		
		P200	N200	P300	P200	N200	P300	P200	N200	P300
F3	Mean	9.2	0.2	8.3	7.8	–3.2	5.3	8.7	–1.6	7.3
	(SD)	(3.4)	(4.0)	(3.2)	(3.4)	(4.1)	(4.1)	(4.8)	(5.3)	(4.9)
Fz	Mean	10.3	0.4	9.7	8.7	–3.2	6.1	10.1	–1.3	9.1
	(SD)	(4.2)	(4.8)	(4.5)	(3.6)	(5.4)	(5.2)	(5.5)	(6.1)	(5.2)
F4	Mean	9.8	1.0	9.8	7.9	–2.2	6.7	9.2	–0.4	9.1
	(SD)	(3.7)	(4.8)	(4.3)	(4.0)	(5.3)	(4.7)	(5.0)	(6.0)	(5.0)
C3	Mean	7.3	0.7	9.0	6.9	–0.37	7.0	7.2	0.6	9.3
	(SD)	(3.1)	(4.2)	(3.5)	(3.1)	(4.6)	(4.4)	(3.4)	(4.6)	(5.3)
Cz	Mean	9.5	2.1	11.0	9.1	0.2	8.9	10.3	1.7	11.5
	(SD)	(2.9)	(0.2)	(4.3)	(4.1)	(5.7)	(5.4)	(4.4)	(5.1)	(5.5)
C4	Mean	8.7	3.0	11.2	7.6	1.3	8.5	8.3	2.8	11.3
	(SD)	(4.0)	(5.1)	(4.1)	(3.6)	(5.7)	(5.2)	(4.2)	(4.9)	(5.4)
P3	Mean	6.1	0.1	7.7	7.8	2.9	8.7	7.0	2.6	9.1
	(SD)	(4.1)	(4.8)	(4.7)	(5.8)	(4.6)	(5.2)	(4.7)	(3.5)	(3.9)
Pz	Mean	8.2	2.2	10.3	8.5	2.7	9.8	8.9	3.9	11.4
	(SD)	(2.6)	(4.3)	(3.6)	(4.5)	(5.3)	(5.2)	(4.1)	(4.5)	(4.1)
P4	Mean (SD)	7.6	2.8	8.1	9.0	3.8	8.3	9.0	4.9	9.3
		(4.7)	(4.7)	(3.8)	(4.9)	(5.2)	(4.1)	(4.3)	(3.7)	(3.3)


No other main effects or interactions were significant for the P200 peak latency and amplitude (all *p* > 0.069).

##### Mismatching stimuli

**FIGURE 5:**
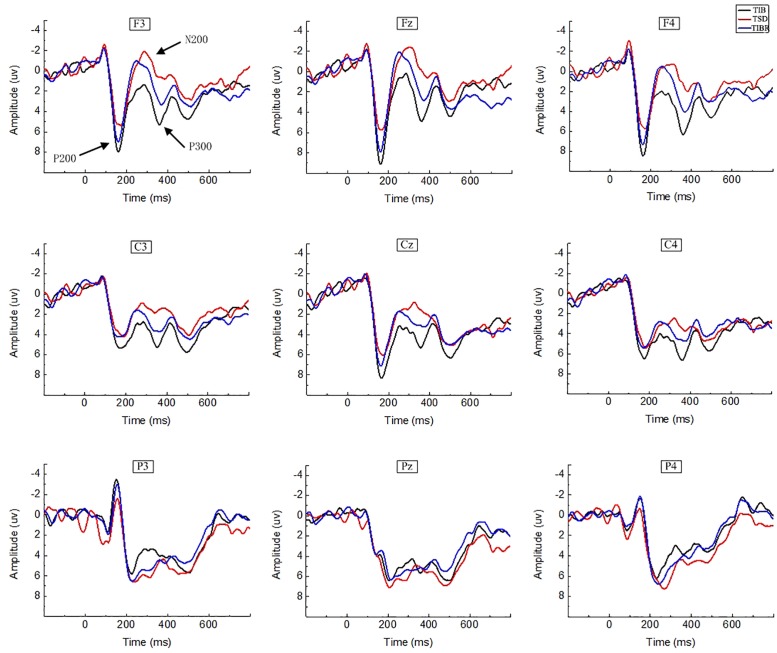
ERP amplitude between sleep conditions for the Mismatching stimuli in working memory. Grand-average ERP (P200, N200, and P300) in the Mismatching stimuli condition across multiple electrode sites after 8 h time in bed (TIB), after 36 h of total sleep deprivation (TSD), and after 8 h time in bed for recovery (TIBR). The channels are ordered left-right and top-bottom as follows: F3, Fz, F4, C3, Cz, C4, P3, Pz, and P4.

**FIGURE 6:**
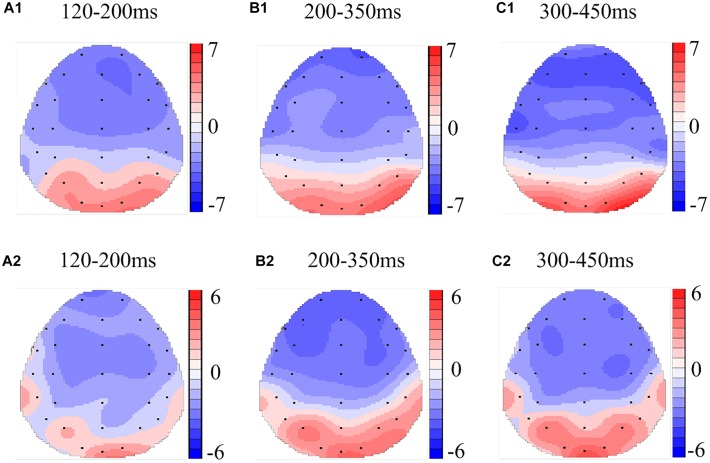
Topographic map of the differences between sleep conditions for Mismatching stimuli of working memory **(A1–C2)**. Head plots of the paired *t*-test approach (two-tailed *t*-test, *p* < 0.05) map the scalp distribution of statistical differences of 36 h of total sleep deprivation (TSD) vs. 8 h time in bed (TIB) **(A1–C1)** and 8 h time in bed for recovery (TIBR) vs. TIB **(A2–C2)**. Color bars indicate *t*-values. **(A1)** P200, 120–200 ms, in TSD vs. TIB. **(B1)** N200, 200–350 ms, in TSD vs. TIB. **(C1)** P300, 300–450 ms, in TSD vs. TIB. **(A2)** P200, 120–200 ms, in TIBR vs. TIB. **(B2)** N200, 200–350 ms, in TIBR vs. TIB. **(C2)** P300, 300–450 ms, in TIBR vs. TIB.

In the Mismatching condition, the P200 wave occurred in the frontal region (Figure 5, F3, Fz, F4). The scalp distribution of statistical differences between the TSD and TIB states (most significant: Fz channel, two-tailed paired *t*-test, *t* = 1.181, *p* < 0.05) implicated the frontal regions as the main neural generators (Figure 6A1) and between the TIBR and TIB states had no significant differences (Figure 6A2).

The main effect of sleep state was significant for the P200 wave [*F*(2,28) = 4.475, *p* < 0.038], indicating that a longer P200 latency occurred in the TSD state than in the TIB (*p* < 0.04) and TIBR states (*p* < 0.05, see Table 4). A significant main effect of the region [*F*(2,28) = 105.092, *p* < 0.001] indicated an increase in activity in the parietal region compared to the central region (*p* < 0.001).

**Table 4:** Grand-average peak latency of the P200, N200, and P300 components in the Mismatching stimuli condition across multiple electrode sites during 8 h time in bed (TIB), after 36 h of total sleep deprivation (TSD), and after 8 h time in bed for recovery (TIBR).

		8 h-TIB			36 h-TSD			8 h-TIBR		
		P200	N200	P300	P200	N200	P300	P200	N200	P300
F3	Mean	162.7	254.1	352.6	173.1	289.2	462.9	160.2	266.0	380.0
	(SD)	(13.8)	(39.1)	(25.7)	(20.4)	(37.4)	(111.4)	(16.2)	(38.2)	(44.8)
Fz	Mean	161.8	259.7	354.2	173.8	291.7	461.5	160.6	258.0	383.4
	(SD)	(11.8)	(26.1)	(23.2)	(21.6)	(38.8)	(120.3)	(15.6)	(37.5)	(51.7)
F4	Mean	161.8	242.5	356.5	178.5	286.7	417.4	161.9	259.4	374.5
	(SD)	(14.0)	(35.9)	(22.9)	(24.4)	(39.8)	(81.5)	(12.6)	(33.5)	(42.7)
C3	Mean	180.8	258.9	373.4	174.2	285.5	442.8	175.4	266.4	393.5
	(SD)	(25.6)	(25.9)	(73.5)	(24.3)	(45.3)	(90.8)	(28.1)	(39.4)	(71.3)
Cz	Mean	177	269.5	367.7	181.7	300.2	472.7	170.8	271.0	399.6
	(SD)	(25.2)	(25.0)	(56.5)	(29.3)	(39.5)	(92.8)	(24.3)	(39.4)	(77.7)
C4	Mean	181.5	269.6	361.7	191.6	298.3	438.1	169.2	280.3	350.8
	(SD)	(27.5)	(36.6)	(42.9)	(26.9)	(36.6)	(90.1)	(28.3)	(34.3)	(36.0)
P3	Mean	213	283.0	413.2	238.7	287.2	438.4	228.6	295.6	373.1
	(SD)	(16.2)	(27.5)	(66.0)	(33.1)	(49.1)	(76.2)	(21.2)	(58.4)	(82.2)
Pz	Mean	190.3	284.2	382.2	228.5	314.3	445.5	195.7	267.0	380.6
	(SD)	(36.4)	(37.0)	(51.7)	(38.3)	(31.2)	(60.9)	(41.2)	(40.9)	(78.0)
P4	Mean	233.9	310.3	382.0	238.2	311.9	410.9	240.3	305.4	403.9
	(SD)	(19.8)	(29.3)	(54.2)	(43.5)	(39.7)	(60.9)	(24.4)	(55.9)	(93.5)


A significant line main effect [*F*(2,29) = 7.085, *p* < 0.009] indicated that the amplitude of the P200 wave was larger in the central region than in the frontal (*p* < 0.023) and parietal regions (*p* < 0.014) for every sleep state (Table 5).

**Table 5:** Grand-average peak amplitude of the P200, N200, and P300 components in the Mismatching stimuli condition during 8 h time in bed (TIB), after 36 h of total sleep deprivation (TSD), and after 8 h time in bed for recovery (TIBR).

		8 h-TIB			36 h-TSD			8 h-TIBR		
		P200	N200	P300	P200	N200	P300	P200	N200	P300
		**P200**	**N200**	**P300**	**P200**	**N200**	**P300**	**P200**	**N200**	**P300**
F3	Mean	9.5	–0.3	6.2	8.3	–4.2	5.4	8.8	–3.0	5.1
	(SD)	(3.9)	(3.7)	(4.2)	(4.2)	(3.5)	(4.5)	(3.6)	(4.2)	(4.4)
Fz	Mean	10.5	–1.5	6.2	8.7	–5.0	5.5	10.0	–4.5	5.1
	(SD)	(4.6)	(4.6)	(4.4)	(4.2)	(4.1)	(4.9)	(4.3)	(4.9)	(5.2)
F4	Mean	9.9	–0.4	7.8	8.5	–3.4	5.7	9.1	–2.9	5.8
	(SD)	(4.7)	(4.2)	(4.2)	(4.2)	(3.7)	(4.1)	(4.1)	(4.9)	(5.2)
C3	Mean	8.0	1.0	6.48	7.1	–1.5	5.5	7.0	–0.6	6.2
	(SD)	(3.1)	(2.8)	(2.8)	(2.6)	(3.1)	(4.2)	(2.8)	(3.4)	(3.9)
Cz	Mean	10.6	1.4	7.4	9.3	–1.5	7.6	9.6	–1.0	6.6
	(SD)	(3.7)	(3.8)	(5.2)	(3.7)	(3.4)	(5.7)	(3.6)	(4.0)	(4.2)
C4	Mean	8.4	1.9	8.2	7.9	0.1	6.7	7.7	0.5	6.6
	(SD)	(3.9)	(3.2)	(4.5)	(2.8)	(3.2)	(4.0)	(3.1)	(3.2)	(4.4)
P3	Mean	7.0	0.8	6.3	9.0	2.5	8.7	7.7	2.4	7.9
	(SD)	(4.1)	(2.8)	(3.2)	(5.7)	(5.3)	(4.1)	(5.4)	(3.9)	(3.1)
Pz	Mean	9.0	1.4	7.7	10.1	2.7	8.8	8.8	2.3	8.4
	(SD)	(2.8)	(2.3)	(3.1)	(4.4)	(4.6)	(3.9)	(3.8)	(3.6)	(2.9)
P4	Mean	7.7	1.5	5.1	9.6	2.3	7.1	8.1	2.1	6.1
	(SD)	(3.7)	(3.5)	(2.7)	(5.4)	(3.3)	(3.4)	(4.5)	(3.7)	(2.6)


There were no other significant main effects or interactions observed in the Mismatching condition (all *p* > 0.129).

#### N200

##### Matching stimuli

Our recordings revealed that the N200 wave occurred 200–350 ms after stimulus onset in the frontal and central regions (Figure 3, F3, Fz, F4, C3, Cz, C4). The scalp distribution of statistical differences between the TSD and TIB states revealed a significant decrease (most significant: F4 channel, two-tailed paired *t*-test, *t* = 1.092, *p* < 0.05) in cortical activity primarily in the right frontal region (Figure 4B1) and between the TIBR and TIB states had no significant differences (Figure 4B2).

A significant main effect of the region [*F*(2,28) = 7.271, *p* < 0.01] revealed that the N200 peak latency was shorter in the frontal than in the parietal region (*p* < 0.0029, see Table 2).

The main effects of region [*F*(2,28) = 4.755, *p* < 0.03] and line [*F*(2,28) = 10.557, *p* < 0.002] were significant in the N200 peak amplitude. These effects were qualified by a significant sleep state × region interaction [*F*(4,26) = 7.216, *p* < 0.005]. Decomposition of this interaction indicated that the N200 responses in the frontal and parietal regions decreased in amplitude in the TSD state compared to the TIB state (*p* < 0.022) as determined by *post hoc* comparisons (Table 3).

No other significant changes were observed in the N200 peak latency or amplitude in the Matching condition (all *p* > 0.146).

##### Mismatching stimuli

The N200 in the Mismatching condition occurred in the frontal region (Figure 5, F3, Fz, F4). The topographic maps of statistical differences in ERP activity between the TSD and TIB states revealed a significant decrease (most significant: F4 channel, two-tailed paired *t*-test, *t* = 3.957, *p* < 0.05) in cortical activity primarily in the frontal region (Figure 6B1) and between the TIBR and TIB states also revealed a significant decrease (most significant: F4 channel, two-tailed paired *t*-test, *t* = 5.089, *p* < 0.001) in the frontal region (Figure 6B2).

A significant main effect of the sleep state [*F*(2,28) = 5.178, *p* < 0.026] revealed that the N200 peak latency in the Mismatching condition was longer in the TSD state than in the TIB state (*p* < 0.035). A significant main effect of the region [*F*(2,28) = 6.398, *p* < 0.05] indicated that the N200 latency was longer in the parietal region than in the frontal region (*p* < 0.009, see Table 4).

The main effects of sleep state [*F*(2,28) = 5.338, *p* < 0.022], region [*F*(2,28) = 6.58, *p* < 0.03], and line [*F*(2,28) = 4.68, *p* < 0.002] were significant for the N200 peak amplitude. These effects were qualified by a significant sleep state × region interaction [*F*(4,26) = 7.216, *p* < 0.005]. This interaction revealed that the N200 amplitude in the frontal and central regions was smaller in the TSD state than in the TIB state in the Mismatching condition. *Post hoc* tests revealed that this difference was significant (*p* < 0.05, see Table 5).

No other significant main effects or interactions were found for the N200 peak latency or amplitude in the Mismatching condition (all *p* > 0.24).

#### P300

##### Matching stimuli

The P300 wave occurred 300–450 ms after stimulus onset in the frontal, central, and parietal regions (Figure 3). The scalp distribution of statistical differences between the TSD and TIB states revealed a significant decrease (most significant: F3 channel, two-tailed paired *t*-test, *t* = 0.813, *p* < 0.05) in cortical activity primarily in the bilateral frontal region (Figure 4C1) and between the TIBR and TIB states had no significant differences (Figure 4C2).

A significant main effect of the sleep state [*F*(2,28) = 5.698, *p* < 0.05] indicated that the P300 peak latency was longer in the TSD state than in the TIB state.

The main effects of sleep state [*F*(2,28) = 11.515, *p* < 0.026], region [*F*(2,28) = 5.010, *p* < 0.05], and line [*F*(2,28) = 11.515, *p* < 0.001] were significant for the P300 peak amplitude. The significant sleep state × region interaction [*F*(4,26) = 3.604, *p* < 0.05] indicated that the P300 amplitude in the Matching condition was smaller in the frontal and central regions in the TSD state than in the TIB state (*p* < 0.050, see Table 5).

No other significant main effects or interactions of the P300 peak latency and amplitude were observed in the Matching condition (all *p* > 0.194).

##### Mismatching stimuli

The P300 wave occurred in the frontal, central, and parietal regions (Figure 5). The topographical maps of statistical differences in ERP activity between TSD and TIB revealed a significant decrease (most significant: Fz channel, two-tailed paired *t*-test, *t* = 0.813, *p* < 0.05) in cortical activity primarily in the frontal region (Figure 6C1) and between the TIBR and TIB states also revealed a significant decrease (most significant: C4 channel, two-tailed paired *t*-test, *t* = 4.109, *p* < 0.002) in the central region (Figure 6C2).

There was a significant main effect of sleep state [*F*(2,28) = 9.86, *p* < 0.004]. The P300 peak latency in the Mismatching condition was longer in the frontal and central regions in the TSD state than in the TIB state (*p* < 0.005, see Table 4).

No other main effects or interactions were significant for the P300 peak latency and amplitude in the Mismatching condition (all *p* > 0.084).

## Discussion

In this study, we combined behavioral data in three sleep states (TIB, TSD, and TIBR) with simultaneous EEG recordings. The analysis revealed that the behavioral data were consistent with decreased WM after sleep deprivation, as shown by the increase in RT and the decrease in accuracy rate. The ERP changes predominantly manifested as a decrease in the amplitudes and an increase in the latencies of the P200, N200, and P300 components. It was found that the changes related to the P200 wave primarily occurred in the frontal regions. After TIBR, the decrease in the above-mentioned ERP components was restored. The results indicate that sleep deprivation severely affects the information processing of phonological WM.

One of the most important findings of this study was the significant decrease in the P200 amplitude after sleep deprivation. P200 appears during the early stage of information processing, and reflects the perception of shape and additional information regarding an object. It has been suggested that the initial features such as contour and shape are registered in the neural structure of the temporal-parietal-occipital region when information is provided to individuals to identify an object and subsequently perform an action (occurring at 0–250 ms) (Cohn et al., 2013). The P200 component might be a part of the early cognitive matching system for information processing and may compare sensory inputs to stored memory (Freunberger et al., 2007). After sleep deprivation, the resources used for sensory inputs and matching processes were reduced. However, two potential reasons could potentially explain the decrease in the P200 wave amplitude: First, information processing was altered, and second, the early identification of information was impaired. Based on the degree of electrophysiological information processing, we support the first view. In previous studies on the effects of sleep deprivation on executive functions (Lee et al., 2003; Gosselin et al., 2005), no such apparent differences in P200 waves were observed. The results also indicated that the P200 component is related to information changes during information processing in WM.

The scalp topographies of the P200 component indicated that the most significant changes in the brain waves associated with sleep deprivation occurred in the frontal region. This finding was consistent with previous functional imaging findings (Drummond et al., 2000; Choo et al., 2005; Chee et al., 2006; Lythe et al., 2012). Based on the theory of WM, the brain regions associated with information change include the parietal and frontal regions, which are closely related to the WM load. Using fMRI techniques and subjects who had undergone 31 h of sleep deprivation, Lythe et al. (2012) found that the lower right parietal lobe activity increased with normal WM load, and the activity of the right lateral prefrontal cortex decreased when the load increased. Although the EEG results did not accurately identify the position, the location of the frontal region indicated the consistency of the neural processing mechanism. The negative changes in the processing of the P200 component in the frontal regions also reflected the effect of sleep deprivation on WM, particularly the impact of workload, which was related to the greater degree of overlap of information processing in WM.

Twenty-four hours of sleep deprivation resulted in declined sustained attention and reduced P300 and Pe amplitudes, demonstrating a gradual breakdown of top-down control, indicating that SD is more detrimental to cognitive functions that are relatively more dependent on mental effort and/or cognitive capacity (Kusztor et al., 2019). The P300 wave primarily reflects the judgment of stimulus consistency in WM, which indicates classical cognitive processing. With regard to the decrease in the P300 wave amplitude, decision-making in the cognitive matching response after sleep deprivation was also impaired to a certain extent (Gosselin et al., 2005; Brauer, 2016). Scalp topographies of the P300 component showed that the activity of this response was primarily concentrated in the frontal and parietal regions. These results further demonstrate that sleep deprivation has an overall impact on WM, rather than on specific information processing stages. Cote et al. (2008) also reported that sleep deprivation affects a more complex stage of information processing. The behavioral response also supported the conclusion that the overall RT was prolonged and the latency of the P300 wave was extended (Brauer, 2016). Presumably, the effect of sleep deprivation on the P300 component might also occur on the basis of response impairment to information change, which supports the conclusion of Donchin and Fabiani (1991) that the P300 component is elicited by the updating of WM content.

After TIBR, the amplitude of the related ERP components was increased. The efficiency of information processing was also restored. This was closely related to the promotion and recovery of memory by sleep. Studies have demonstrated that the recovery of impaired memory during sleep also has an effect on newly formed memories (Mu et al., 2005; Rasch and Born, 2007). TIBR after sleep deprivation differs from general sleep, and studies have indicated that the depth of sleep might increase. However, due to the influence of sleep debt, the degree of recovery is limited (Mander et al., 2010; Boardman et al., 2017). From a physiological point of view, restorative sleep did not restore baseline levels, indicating that after prolonged sleep deprivation, longer TIBR is required to restore the impaired WM.

The study has some limitations. First, the n-back model was used to study the effects of sleep deprivation on WM. However, no 0-back or 1-back contents were included in the study. Therefore, it is necessary to be cautious in the interpretation and inference of the changes in workload. Second, with respect to the implementation of the project, as no female subjects were included and we did not consider the differences between children and the elderly, the extrapolation of the results requires caution. Third, brain network analysis combined with EEG recordings and brain imaging may facilitate further interpretation of the results. Fourth, we did not monitor sleep architecture during the present study, although this information can help analyze the sleep status of subjects; this should be explored in future research. Future studies should also consider examining the association between EEG during the previous night’s sleep (e.g., slow-wave activity) and the subsequent ERP.

This study examined the electrophysiological features of the changes in WM under the influence of sleep deprivation. Notable changes were identified in the P200 component, which reflects the change in information, is one of the WM processing components greatly influenced by TSD. We found that 8 h of TIBR were not sufficient to recover from the effect of 36 h of TSD on WM. This study provides ERP evidence useful to understand the mechanism behind the effects of sleep deprivation on WM.

## Ethics Statement

This study was carried out in accordance with the recommendations of “PLA Army General Hospital” with written informed consent from all subjects. All subjects gave written informed consent in accordance with the Declaration of Helsinki. The protocol was approved by the PLA Army General Hospital and Beihang University.

## Author Contributions

LZ and YS designed the research. LZ produced the results. LZ wrote the manuscript. YS, ZL, CL, YC, and QZ read and approved the final manuscript.

## Conflict of Interest Statement

The authors declare that the research was conducted in the absence of any commercial or financial relationships that could be construed as a potential conflict of interest.

## Abbreviations

ERPs: event-related potentials
PWMTB: pronunciation WM two-back task
TIB: time in bed
TIBR: time in bed for recovery
TSD: total sleep deprivation
WM: working memory
